# The Functional Anatomy of Psychomotor Disturbances in Major Depressive Disorder

**DOI:** 10.3389/fpsyt.2015.00034

**Published:** 2015-03-10

**Authors:** Benny Liberg, Christoffer Rahm

**Affiliations:** ^1^Department of Psychiatry, Melbourne Neuropsychiatry Centre, The University of Melbourne, Melbourne, VIC, Australia; ^2^Division of Medical Imaging and Technology, Department of Clinical Science, Intervention and Technology (CLINTEC), Karolinska Institutet, Stockholm, Sweden; ^3^Unit of Metabolism, Department of Medicine Huddinge, Karolinska Institutet, Stockholm, Sweden

**Keywords:** psychomotor performance, major depressive disorder, neuroimaging, frontal lobe, basal ganglia, monoamines

## Abstract

Psychomotor disturbances (PMD) are a classic feature of depressive disorder that provides rich clinical information. The aim our narrative review was to characterize the functional anatomy of PMD by summarizing findings from neuroimaging studies. We found evidence across several neuroimaging modalities that suggest involvement of fronto-striatal neurocircuitry, and monoaminergic pathways and metabolism. We suggest that PMD in major depressive disorder emerge from an alteration of limbic signals, which influence emotion, volition, higher-order cognitive functions, and movement.

## Introduction

Psychomotor signs are a classic feature of major depressive disorder that already attracted attention over a century ago (1). Emil Kraepelin gave a vivid and still valid description of psychomotor disturbances (PMD) in his chapter on general symptomatology in *Lehrbuch des Psychiatrie*, 1907: “The psychomotor retardation, which is the most important disturbance in the depressed states of manic-depressive insanity, is probably due to a […] increase in resistance […] In spite of every apparent exertion, the patients cannot utter a word or at best answer only in monosyllables, and are unable to eat, stand up, or dress. As a rule they clearly recognize the enormous pressure lying upon them, which they are unable to overcome” (2).

Psychomotor disturbances in depressive disorder can be broadly classified in to four subgroups of symptoms and signs based on three available clinical rating scales designed to characterize them [CORE, motor agitation and retardation scale (MARS), Widlöcher scale] (3–5): retardation, agitation, non-interactiveness, and mental slowing (Table 1). The symptoms and signs of PMD therefore entail a wide range of brain functions including motor performance, executive function, volition, and drive. These provide rich clinical information (i.e., diagnostic subgroup, prognosis, treatment) (6, 7).

**Table 1:** **Psychomotor signs in major depressive disorder**.

Subgroup of psychomotor disturbances	Example
Retardation	Slowed movements (motor slowness), facial immobility (lack of facial expressivity, downcast gaze, reduced voice volume, slurring of speech), body immobility (immobility of trunk/proximal limbs), postural slumping (postural collapse), delay in motor activity, delay in responding verbally (delayed speech onset), slowing of speech rate (monotone speech), abnormal gait
Agitation	Frightened apprehension (static facial expression, abnormal staring, increased blinking, erratic eye movement), facial agitation (movement/tension in mouth), motor agitation (increased axial truncal movement), stereotyped movements (tension in fingers and hands, hand movement, foot/lower leg movement), verbal stereotypy
Non-interactiveness	Response to social cues, emotional responsiveness, inattentiveness, poverty of associations, spontaneous speech, length of verbal responses
Mental slowing	Language and verbal flow, variety of themes spontaneously approached, richness of associations, subjective experience of ruminations, fatigability, perception of flow of time, memory, concentration, interest in habitual activities

No previous review has focused specifically on neuroimaging findings related to PMD in major depressive disorder. The aim of this narrative review is to characterize the functional anatomy of PMD in major depressive disorder by summarizing findings from human neuroimaging studies that probe structure, function, neurochemistry, and connectivity.

## Structural Neuroimaging

Structural aberrations in white matter are the most prominent structural neuroimaging findings associated with PMD in depressive disorder.

White-matter alterations (hyperintensities, WHI; and white-matter fiber integrity), are one of the most reproduced findings in mood disorders. White-matter hyperintensities (WHIs) are radiological hyperintense regions of white matter with elusive etiology in MRI images. They are primarily associated with late-life depression, but are also more common in major depressive disorder in younger age groups. The extent of WHIs correlates with illness severity, poor treatment response, and decreased psychomotor speed on several neuropsychological tests (8). White-matter tissue broadly comprises glial cells with myelin surrounding axons. Currently, the general understanding is that the WHIs alterations observed in depression arise from small vessel disease that lead to disruption of white-matter pathways (9). However, other disease mechanisms involving white-matter tissue may also lead to disruptions of specific neurocircuits and lead to psychiatric symptoms such as PMD (10).

White-matter fiber integrity can be assessed with diffusion-weighted imaging. One study by Walther et al. (11) who specifically addressed psychomotor functioning in depressive disorder used diffusion-weighted magnetic resonance imaging and actigraphy – an objective measure of the general activity level in an individual. It showed that lower activity levels correlate with measures of differential myelinization in the frontal lobe and posterior cingulate region, and that there is a negative correlation between the same measures in the white matter beneath the primary motor cortex and in the parahippocampal region. The authors conclude that changes in psychomotor function in depressive disorder may be linked to changes in white matter in motor regions. Bracht et al. used diffusion-weighted imaging to investigate white-matter microstructure in relation to PMD. They found a positive association between decreased physical motor activity and alterations in paralimbic and motor midline regions not only involved in volitional movement but also involvement of ascending mesocortical dopamine pathways in clinical states with prominent PMD (12, 13).

To this date, few studies have investigated the relation between gray matter volume and PMD in major depressive disorder. Current findings involve volume reductions in several pre-executive parts of the motor system. One volumetric study showed that thinning of the right presupplementary motor cortex (pre-SMA) is associated with impaired performance on a motor learning test (14). The pre-SMA is a part of the mesial premotor cortex that advances signals from the prefrontal regions, engaged in higher-order cognitive functions. In studies measuring subcortical volumes and regional shape alterations, no significant associations could be found between performance on a psychomotor task (trail making test variations) and the volumes of striatum, pallidum, and thalamus in depressed subjects (15, 16). Another study found that reduced caudate nucleus volumes predicts decreased psychomotor speed in depressed subjects >50 years old (17).

Only one study, using CT, has assessed cerebrospinal fluid space size. This study found that the size of the third ventricle was associated with clinical ratings of psychomotor retardation (18).

## Functional Neuroimaging

Blood–oxygen-level-dependent (BOLD) functional magnetic resonance imaging (fMRI) is currently the most prevalent method for studying neural activation patterns during experimental tasks in patients with depressive disorder. A few research teams have specifically addressed PMD using fMRI and experimental motor tasks, clinical ratings of psychomotor disturbance, or motor physiology metrics (i.e., actigraphy, reaction time). Two types of studies have been employed – *task* and *non-task* based studies. Naismith et al. (19) used a motor sequence task (button press response) to study motor learning, and found increased activation of lateral prefrontal cortex, superior temporal regions, and the cerebellum. Caligiuri et al. (20, 21) studied motor execution using a manual reaction time task, and found increased activation during movement in the primary motor cortex, alongside motor asymmetry. Five other studies investigated motor speed using different finger-tapping variations (22–27), and suggest an increased activation in both motor and paralimbic regions, and with altered fronto-striatal coupling among patients. One non-task, resting-state study, by Yao et al. (28) corroborates the hyperactivation of paralimbic regions in patients.

## Electroencephalography

Electroencephalography (EEG) is used to study power amplitude of particular frequency spectrums, hemisphere asymmetry, and chronometric features of cortical neural activation. PMD have been associated with greater variability and increased amplitudes in the delta (<4 Hz) and theta (4–7 Hz) spectrum, but not with hemisphere asymmetry (29). The post-imperative negative variation is a metric related to frontal lobe function, and has been associated with psychomotor slowing in a choice reaction task (30). Another frontal metric (P300) has also been correlated positively correlated with PMD (31). Interestingly, this study also showed that only clinical ratings more focused on PMD than the Hamilton depression ratings scale (HDRS) predicted P300 latency. In a group of patients receiving electroconvulsive treatment, clinical ratings of PMD were positively correlated with frequency decreases during initial improvement, whereas the reverse relationship was found during the later partial remission phase (32). One study by Nieber et al. (33) showed a positive correlation between decreased frequencies in particular regions of the theta and alpha (7–13 Hz) spectrum and overall retardation, with motor retardation, in particular. In that study, increased frequency in particular regions of in the alpha and beta spectrum was negatively correlated with PMD. Error-related negativity and positive-negativity are metrics associated with anterior and posterior cingulate cortex function, respectively (34, 35). These metrics have been associated with a slowing of psychomotor performance in subjects during action monitoring, but only positive-negativity differentiated patients and controls (36).

## Molecular Neuroimaging

Single-photon emission tomography (SPECT), positron emission tomography (PET), and arterial spin labeling (ASL) are the three molecular neuroimaging methods that have been used to study PMD. These three methods measure regional cerebral blood flow, glucose metabolism, oxygen consumption, or synaptic transmission factors. Walther et al. (37) used ASL and actigraphy to measure the correlation between regional cerebral blood flow and general motor activity outside of the scanner environment in depressed subjects. The study showed a positive correlation between physical activity and blood perfusion in the right orbitofrontal cortex, and a negative correlation with left supplementary motor area perfusion. The available evidence from PET and SPECT studies also suggests that PMD in depression are associated with decreased DLPFC metabolism (38–40), increased ACC metabolism (41–43), and a lower dopaminergic tone and altered metabolism in striatal regions (41, 42, 44–47). However, a SPECT study by Graff-Guerrero et al. (48) failed to reproduce these associations between clinical rating of PMD and cerebral blood flow. One longitudinal study also suggests that improvement of psychomotor slowing is associated with increased activation in the dorsal ACC (49).

## Transcranial Ultrasound

Hypo- or hyperechogenicity measured by transcranial sonography *in vivo* reflect changes in tissue impedance, likely due to alterations of microarchitecture such as shifts in cell density, changes in interstitial matrix composition, or alterations of fiber tract integrity (50, 51). Those transcranial ultrasound studies that have investigated PMD in major depression have focused on the serotonergic raphe nuclei and the dopaminergic substantia nigrae. A significantly reduced echogenicity of the mesencephalic midline raphe nuclei has been reported in depressed subjects (52). Hypoechogenicity of the raphe nuclei can be found in 50–70% of unipolar depressed subjects compared to 10% in healthy subjects (53). Hypoechogenicity of the raphe nuclei of the brain stem is associated with better treatment response to serotonin reuptake inhibitors (54) and with symptom severity in suicidal ideation (55). One study could not find any association between echogenicity of the raphe nuclei and PMD (51), another found a positive correlation with the degree of psychomotor retardation (56), and a third a negative correlation with psychomotor retardation (54). Hoeppner et al. showed that substantia nigra echogenic size correlates with motor asymmetry and reduced verbal fluency in unipolar depression. In that study, the association was stronger in patients ≥50 years, and in patients with reduced brain stem raphe nuclei hypogenicity (57).

## Conclusion

In this review, we summarize the literature on the functional neuroanatomy of PMD in major depressive disorder (Table 2). Despite the clinical importance of PMD, we found relatively few studies. Indeed, the motor system has been relatively neglected in brain imaging studies of psychiatric disorders in general (58). We conclude that structural alterations that correlate with PMD have been found in gray- and white-matter regions within several nodes of cortico-subcortical circuits. Findings in functional neuroimaging studies show involvement of the same neurocircuitry nodes (along with their white-matter connections) as in structural neuroimaging studies, and further that limbic influences on the motor system may be important in the emergence of PMD. EEG studies suggest that frequency variations across many spectra, and an involvement of the frontal cortex, anterior, and posterior cingulate cortex, are associated with PMD. The molecular neuroimaging correlates of PMD resemble the functional anatomy of major depression described with functional and structural methods, but in addition also implicate disrupted monoamine transmission in PMD. The few available studies that use transcranial ultrasound primarily show an association between PMD and echogenic features of the substantia nigra, which then corroborates molecular neuroimaging findings of disrupted dopamine transmission.

**Table 2:** **Neuroimaging findings and their correlation to psychomotor disturbances**.

	Study	*N*	Diagnosis	Method	Measure	Finding
Structural CT and MRI	Hickie et al. (8)	39	MDD	MRI (WMH)	Mean decision time	↑ White-matter hyperintensities
	Walther et al. (11)	21	MDD	DTI (FA)	Actigraphy	↓ White-matter in motor regions
	Bracht et al. (12)	21/21	MDD	DTI (FA)	Actigraphy	↓ White-matter in ACC and midline motor regions connected with PFC
	Bracht et al. (13)	22/21	MDD	DTI (FA)	Clinical features of PMD	↓ White-matter in medial forebrain bundle
	Exner et al. (14)	9	MDD	MRI (ROI)	Serial reaction time task	↓ pre-SMA volume
	Liberg et al. (15)	27	BPD	MRI (ROI, shape)	Trail Making Tests, reaction Time	No significant findings in the striatum, pallidum, and the thalamus
	Liberg et al. (16)	20	BPD	MRI (ROI, shape)	Trail Making Tests	No significant findings in the striatum, pallidum, and the thalamus
	Naismith et al. (17)	47	MDD	MRI (ROI)	Trail Making Test A	↓ Right caudate volume
	Schlegel et al. (18)	44	MDD	CT, ventricle size	Bech–Rafaelsen Melancholia Scale	↑ Lateral ventricle size
fMRI	Naismith et al. (19)	19/20	MDD	Task-based fMRI	Motor sequencing task	↑ Middle frontal gyrus, superior temporal gyrus, and cerebellum
	Caligiuri et al. (20)	24/13	BPD	Task-based fMRI	Manual reaction time task	↑ Right primary motor cortex in patients
	Caligiuri et al. (21)	18/13	BPD	Task-based fMRI	Manual reaction time task	↑ Left primary motor area in patients. Motor asymmetry in patients with a failure to suppress right hemisphere activation during movement
	Marchand et al. (22)	10	BPD	Task-based fMRI	Finger-tapping	↑ Right anterior cingulate cortex and medial frontal gyrus (euthymia > depression)
	Liberg et al. (24)	9/12	BPD	Task-based fMRI	Finger-tapping	No significant findings
	Liberg et al. (25)	9/12	BPD	Task-based fMRI	Finger-tapping, Motor imagery, CORE, AS-18	↓ Primary motor cortex, lateral ventral premotor cortex in relation to clinical ratings. ↑ Medial posterior parietal cortex during motor imagery. ↑ Fronto-parietal regions, and insular cortex, during motor execution
	Liberg et al. (26)	13/13	MDD	Task-based fMRI	Finger-tapping	↓ Fronto-striatal coupling between cingulate motor area and putamen. ↑ Left cingulate motor area. ↑ Functional coupling and clinical ratings
	Marchand et al. (27)	14/15	BPD	Task-based fMRI	Finger-tapping	↑ Left pre- and post-central gyrus, bilateral cingulate, right striatum, and left striatum, in patients
	Yao et al. (28)	22/22	MDD	Resting-state fMRI	HDRS	↑ Regional homogeneity in right posterior cingulate cortex and right insula
EEG	Nyström et al. (29)	25	MDD	EEG power spectrum analysis	Comprehensive Psychopatho-logical Rating Scale	↑ Delta-, theta-amplitude, and variability
	Thier et al. (30)	11/11	MDD	ERP	Serial choice reaction task	↑ Post-imperative negative variation
	Schlegel et al. (31)	36	MDD	ERP	Bech–Rafaelsen Melancholia Scale	↑ P300 latency
	Silfverskiöld et al. (32)	21	MDD	Global EEG frequency	Rating Scale for Affective Symptoms	↓ Acute effects ↑ Non-acute effects
	Nieber et al. (33)	63	MDD	EEG power spectrum analysis	Bech–Rafaelsen Melancholia Scale	↑ Slow activity ↓ Fast activity
	Schrijvers et al. (36)	26	MDD	ERP, Eriksen Flanker’s Task	Salpêtrière Retardation Rating Scale	↑ Error-related negativity potentials
Molecular neuroimaging	Walther et al. (37)	20/19	MDD	ASL	Wrist actigraphy	↑ Right orbitofrontal cortex, ↓ left SMA
	Bench et al. (38)	40	MDD	PET	HDRS	↓ rCBF in left DLPFC, left parietal cortex
	Dolan et al. (39)	40	MDD	PET	HDRS	↓ rCBF in left DLPFC
	Videbech et al. (40)	42	MDD	PET	HDRS	↓ rCBF in DLPFC and OFC
	Milak et al. (41)	298	MDD	FDG-PET	HDRS	↑ Metabolism in the cingulate gyrus, thalamus, and basal ganglia
	Dunn et al. (42)	58	MDD	FDG-PET	Beck’s Depression Inventory	↓ Metabolism in right insula, claustrum, anteroventral caudate/putamen, and temporal cortex.
						↑ Metabolism in ACC
	Mayberg et al. (43)	13	MDD	99mTc-SPECT	Finger-tapping	↑ rCBF in paralimbic cortex (frontal and temporal) and prefrontal
	Meyer et al. (44)	9/21	MDD	RTI-32-PET	Finger-tapping	↓ Dopamine transporter binding potential in striatum
	Meyer et al. (45)	21	MDD	Raclopride PET	Finger-tapping	↑ Dopamine D2 receptor binding potential in the putamen
	Ebert et al. (46)	20	MDD	IBZM-SPECT	–	↑ Striatal IBZM-BP
	Perico et al. (47)	15	MDD	99mTc-SPECT	HDRS	↑ Left premotor cortex and right anterior medial orbitofrontal cortex metabolism
	Graff-Guerrero et al. (48)	14	MDD	99mTc-SPECT	HDRS	No significant correlation between retardation and CBF
	Brody et al. (49)	39	MDD	FDG-PET	HDRS	Improvement in psychomotor symptoms is associated with metabolism in dorsal ACC
Transcranial sonography	Berg et al. (51)	31	PD with MDD	Ncl raphe	Columbia University Rating Scale	No significant correlation
	Walter et al. (53)	55	MDD	Ncl raphe, substantia nigra	Unified Parkinson’s Disease Rating Scale (Motor part)	↓ Raphe echogenicity, ↑ Substantia nigra echogenecity
	Walter et al. (54)	52	MDD	Ncl raphe	Motor Retardation and Agitation Scale	↑ Raphe echogenecity
	Becker et al. (56)	30	PD with MDD	Ncl raphe	Columbia University Rating Scale	↓ Raphe echogenecity
	Höppner et al. (57)	45	MDD	Substantia nigra	Finger-tapping (motor asymmetry), verbal fluency	↑ Substantia nigra echogenic size

*ACC, anterior cingulate cortex; AS-18, affektiv skattningsskala 18 (59); ASL, arterial spin labeling; BP, binding potential; BPD, bipolar disorder depression; CT, computed tomography; DTI, diffusion tensor imaging; DLPFC, dorsolateral prefrontal cortex; EEG, electroencephalography; ERP, event-related potentials; FA, fractional anisotropy; FDG-PET, fluorodeoxyglucose positron emission tomography; fMRI, functional magnetic resonance imaging; HDRS, Hamilton Depression Rating Scale; IBZM, iodobenzamide single-photon emission computed tomography; MDD, major depressive disorder; MRI, magnetic resonance imaging; OFC, orbitofrontal cortex; PD, Parkinson’s disease; PET, positron emission tomography; ROI, region of interest; rCBF, regional cerebral blood flow; RTI-32, (1R-2-exo-3-exo)-8-methyl-3-(4-methylphenyl)-8-azabicyclo[3.2.1]octane-2-carboxylate; SMA, supplementary motor area; SPECT, single-photon emission computed tomography; 99mTc, Technetium-99*.

Structural and functional neuroimaging studies suggest that PMD involve alterations in large-scale cortico-striato-thalamo-cortical neurocircuits, and in particular fronto-striatal subdivisions. Findings from transcranial ultrasound, and molecular neuroimaging studies, suggest a putative underlying factor for these alterations in the form of disrupted influence of ascending dopamine tracts that emanate from deeper midbrain nuclei. This notion also fits with the broader picture of a depressive disorder with psychomotor disturbances, which also include alterations in cognitive function, drive, and emotional expression – phenomena that also map onto ascending monoamine tracts with targets in the frontal lobe. Taken together, the broad picture suggests that PMD in major depressive disorder emerges from altered limbic signals at the interface of emotion, volition, higher-order cognitive function, and movement.

Our review shows that PMD is an emerging field of research that has kept growing since over 20 years. However, the currently available studies also preclude firmer evidence when evaluated in the context of general research methodology. Most studies are cross-sectional, have <25 participants, and have not been reproduced. Furthermore, a wide variety of clinical psychomotor measures have been used. Thus, information about the anatomical specificity of PMD from future studies could be improved by the use of objective measurements of motor performance (i.e., finger-tapping, actigraphy) when investigating the different dimensions of PMD delineated by current clinical measurements (i.e., CORE, MARS), and using rating scales that probe PMD specifically. Further studies would also benefit from longitudinal experimental designs that disentangle the effects of brain changes on the functional components of PMD, and assess differences across neuropsychiatric disorders.

## Conflict of Interest Statement

The authors declare that the research was conducted in the absence of any commercial or financial relationships that could be construed as a potential conflict of interest.
